# Epidemiological Analysis From 2018 to 2020 in China and Prevention Strategy of Porcine Circovirus Type 2

**DOI:** 10.3389/fvets.2021.753297

**Published:** 2021-11-16

**Authors:** Ying Huang, Xianghong Chen, Yunzhi Long, Liu Yang, Wenbo Song, Jinjin Liu, Qianqian Li, Gong Liang, Daobing Yu, Chao Huang, Xibiao Tang

**Affiliations:** Diagnostic Center Department, Wuhan Keqian Biology Co., Ltd., Wuhan, China

**Keywords:** epidemiological analysis, PCV2b Cap, PCV2d Cap, homologous protection, heterologous protection

## Abstract

Porcine circovirus type 2 (PCV2) is one of the smallest known animal viruses and is the main pathogen of PCV-associated diseases (PCVAD). Epidemiological surveillance results have shown that the PCV2 infection rate is on the rise in China, thus, PCV2 disease prevention and control has become a huge challenge for the Chinese swine industry. We collected clinical samples from multiple different provinces in China from 2018 to 2020 and found that the positive rate of PCV2 was 53% (3619/6872), identity between the cloned 62 *ORF2* genes was 84.4–100% and identity between the cloned 62 ORF2 sequences and reference sequence was 72.9–99.8%. Genetic evolution analysis found that PCV2d accounted for 79% (49/62 samples), PCV2a for 12.9% (8/62 samples), PCV2b for 8% (5/62 samples), and PCV2c and PCV2e genotypes were not found. However, most commercial PCV2 subunit vaccines are based on the PCV2a genotype, and there are very few vaccines based on PCV2b or PCV2d. Therefore, the homologous and heterologous protection ability of PCV2b and PCV2d Cap proteins based on the baculovirus against the PCV2b and PCV2d infections was evaluated, which is expected to design and develop excellent PCV2 protein vaccine candidates. This study found that both PCV2b and PCV2d Cap proteins can increase the level of humoral immunity and cellular immune response in mice. Importantly, both PCV2b and PCV2d cap proteins can provide homologous and heterologous protection against the PCV2b and PCV2d viruses. Overall, this study provides a reference for the prevention and control of PCVAD in mainland China and the development of PCV2 vaccines.

## Introduction

Porcine circovirus (PCV) belongs to the family *Circoviridae* and genus *Circovirus* (1). There are three major genotypes of PCV: PCV PCV1, PCV2, and the newly found PCV3 (2). Since the late 1990s, PCV2 has emerged as a major pig pathogen around the globe, easily infecting pigs 4–16 weeks of age, and mainly causes typical clinical appearances, consisting of post-weaning multisystemic wasting syndrome (PMWS), porcine dermatitis nephropathy syndrome (PDNS), reproductive disorders, and respiratory diseases. PCV2 mainly destroys the lymphatic system, leading to defects in the body's immune function, which may cause susceptibility to other diseases, such as porcine parvovirus, pseudorabies virus, and porcine streptococcus (3).

The PCV2 genome is composed of a single strand of circular closed DNA with a length of approximately 1.7 kb. The virus replicates in a rolling circle, producing double-stranded DNA replicas. A study found that the whole genome of PCV2 contains 11 open reading frames (ORFs) (4). The research objects are mainly concentrated in ORF1, ORF2, ORF3, and ORF4. ORF1 encodes a viral replication-related protein with a length of 945nt, which is located on the sense strand of the genome, and its primary transcript is alternatively spliced to produce two variants of rep (35.7 kDa) and rep' (20 kDa) (5). ORF2 is located on the antisense strand of the genome and encodes the Cap protein with 233–236 amino acids. It is the only Cap protein of the virus with a size of about 27.8 kDa. The Cap protein is also an important immunogenic protein of the virus and can induce the body to generate neutralizing antibodies against PCV2 (6). ORF3 is reverse-encoded in ORF1 and encodes a non-structural protein of the virus, which encodes 104 amino acids (11.9 kDa). Studies have shown that the ORF3-encoded protein induces cell apoptosis *in vitro* by activating the caspase 3 and 8 pathways (7). ORF3 is related to the pathogenicity of the virus in animals, and the pathogenicity of PCV2 mutants lacking ORF3 is lower than that of wild-type PCV2 in mice and pigs (7). The protein encoded by ORF4 is not necessary for virus replication. It can inhibit caspase activity and regulate the role of CD4+ and CD8+ lymphocytes during PCV2 infection (8).

Based on the PCV2 whole genome or PCV2-ORF2 sequence system evolution analysis, PCV2 mainly has five genotypes; namely, PCV2a, PCV2b, PCV2c, PCV2d, and PCV2e, among which PCV2a/2b/2d subtypes have attracted wide attention from researchers (9). Before 2003, the virus was dominated by the PCV2a subtype. From 2004 to 2013, it was dominated by the PCV2b subtype. After 2013, it was dominated by the PCV2d subtype, and other subtypes still existed in pig herds (10). The PCV2c subtype is only found in Denmark and Brazil and the number of cases is small. Retrospective studies have shown that PCV2d was first discovered in Switzerland in 1998. Chinese scholars named the newly discovered PCV2 subtype PCV2d for the first time in 2010, and it is now the main popular subtype of PCV2 (11). PCV2e was first discovered in Mexico, and subsequent retrospective studies have shown that this virus subtype also exists in the United States. The earliest gene sequence information was released in 2006 (12). PCV2 can cause multiple disease syndromes in infected pigs, such as PMWS, PDNS, porcine respiratory disease complex, sow reproduction disorders, nervous system damage, and proliferative and necrotizing pneumonia, these disease syndromes are collectively called PCV diseases (PCVD) in Europe and PCV-associated diseases, PCVAD) in North America (13). Early research suggests that the epidemic strain has changed from PCV2a to PCV2b and gradually formed an epidemic worldwide since 2003 (14). A previous study found that 55% (22/40 samples) of their PCV2-positive samples were PCV2d (15), which implied that PCV2d is an ongoing and dominant PCV2 subtype in China. Subsequently, another study also found that PCV2d made up most of the PCV-positive isolates (68.2%; 45/66 samples) in southern China from 2011 to 2012 (16). These data provide evidence that a dynamic genotype shift occurs from PCV2b to PCV2d and that PCV2d dominates worldwide, including in America, Europe, Korea, and Thailand (10, 17, 18).

Although vaccination can effectively reduce the prevalence of PCV2 and alleviate the clinical signs of PCVAD, its continuous mutation through point mutations and genetic recombination causes concern that it may escape vaccination. The PCV2 vaccine needs to be updated regularly, including switching to a new strain. Therefore, obtaining genetic variation information related to PCV2 in pig herds can be crucial for vaccine development and the prevention of PCVAD. The main purpose of this study was to design and develop excellent candidate protein vaccines on the basis of the genetic variation and phylogenetic characteristics of PCV2 in China from 2018 to 2020.

## Materials and Methods

### Ethics Statement

This study was conducted in accordance with the Chinese Laboratory Animal Administration Act of 1988. All animal experimental procedures were performed in accordance with the “Guidelines for Experimental Animals” of the Ministry of Science and Technology (Beijing, China). Animal experiments in this study were subject to approval by the Hubei Province Science and Technology Department, concerning experimental animal ethics. The experiments were carried out under the supervision and inspection of the Scientific Ethical Committee for Experimental Animals of Huazhong Agricultural University, Wuhan, China. The field studies did not involve endangered protected species.

### Samples

The 6,827 clinical samples, including those of the lungs, kidneys, lymph nodes, and spleen, were collected from commercial farms in China (including different provinces) from 2018 to 2020 and stored at −80 °C. Next, the DNA was extracted according to the manufacturer's instructions of the viral DNA extraction kit (TransGen, China) and stored at −80 °C.

### Identification of PCV and Cloning and Sequencing of the ORF2 Gene

Primers were designed based on the PCV2 sequence registered in GenBank (GenBank accession number: AY424401.1). Primers F1 and R1 were used to detect the PCV and primers F2 and R2 were used to amplify the ORF2 gene of the PCV. The primers are shown in Table 1.

**Table 1:** Primer pairs used in this study.

**Primer name**	**Sequence in 5^′^-3^′^ direction**
F1	CACGGATATTGTAGTCCTGGT
R1	CGCACCTTCGGATATACTGTC
F2	GAGGATTACTTCCTTGGTATTTTGG
R2	ATTCTTCTTGCTGGGCATGTTG
F3-2b	AAGGATCCATGACGTATCCAAGGAGGCGTTACC
R3-2b	AAGAGCTCTTAAGGGTTAAGTGGGGGGTCTTTA
F4-2b	CACCATGGATGACGTATCCAAGGAGGCGTTACC
R4-2b	CTGGTACCTTAAGGGTTAAGTGGGGGGTCTTTA
F3-2d	AAGGATCCATGACCTACCCTCGCCGCC
R3-2d	AAGAGCTCTTACTTAGGGTTCAGAGGA
F4-2d	CACCATGGATGACCTACCCTCGCCGCC
R4-2d	CTGGTACCTTACTTAGGGTTCAGAGGA
PCV2_ORF2_F	CGGATATTGTAGTCCTGGTCGTA
PCV2_ORF2_R	CCTGTCCTAGATTCCCCTATTGATT
PCV2_ORF2_Probe	FAM-CTAGGCCTACGTGGTCTACATTTC-TAMRA

To detect PCV-positive samples, the PCR reactions were prepared in a total volume of 20 μL:10 μL of 2 × Easy Taq PCR SuperMix (TransGen Biotech, Beijing, China), 1 μL of primer pairs (F1 and R1), 6 μL of ddH_2_O, and 2 μL of DNA template. PCR amplification was initiated at a predenaturation stage of 95 °C for 5 min, followed by 35 cycles of denaturation at 95 °C for 30 s, annealing at 55 °C for 30 s extension at 72 °C for 30 s, and 10 min extension at 72 °C. Amplified PCR products were subjected to agarose gel electrophoresis.

Next, 62 positive PCV samples were randomly selected for amplification of the PCV ORF2 gene. The PCR reactions were prepared in a total volume of 20 μL:10 μL of 2 × Easy Taq PCR SuperMix, 1 μL of primer pairs (F2 and R2), 5 μL of ddH_2_O, and 3 μL of DNA template. PCR amplification was initiated at a predenaturation stage of 94 °C for 5 min, followed by 35 cycles of denaturation at 94 °C for 30 s, annealing at 53 °C for 40 s, extension at 72 °C for 30 s, followed by an extension at 72 °C for 10 min. The PCR product was then sent to Sangon Biotechnology (Beijing, China) for sequencing.

### Sequence Analysis

The general analysis, including the trimming and assembling of raw sequencing data, conversion into reverse complement sequences, and conceptual translation into protein sequences, was performed using CLC Main Workbench 7 software (Qiagen). The final length of the analysis sequence was 482 bp, and 20 PCV2 reference strain whole genomes and PCV2 ORF2 sequences were downloaded from GenBank. The information of 20 PCV2 strain reference sequences is shown in Table 2. Multiple alignments of nucleotides and deduced amino acid sequences diversity analysis were performed by MEGAlign software. The phylogenetic tree was constructed by the neighbor-joining method using the maximum composite likelihood model with MEGA v6 software. The reliability of the cluster separated in the tree was evaluated by performing 500 bootstrap replicates.

**Table 2:** 20 PCV2 reference strains used for sequence alignment.

**GenBank**	**Geographic origin**	**Genotype**	**GenBank**	**Geographic origin**	**GenBank**
AF118097	Canada	PCV2a	EU148503	Denmark	PCV2c
AF055392	Canada	PCV2a	EU148504	Denmark	PCV2c
DQ397521	United States	PCV2a	EU148505	Denmark	PCV2c
EU450591	Korea	PCV2a	KJ094599	Brazil	PCV2c
AF055394	France	PCV2b	HM038017	China	PCV2d
HM038026	China	PCV2b	HQ395032	China	PCV2d
KP231116	Italy	PCV2b	JX535296	United States	PCV2d
GU799576	United States	PCV2b	AY181946	China	PCV2d
KT870147	United States	PCV2e	KT870146	United States	PCV2e
KT795289	United States	PCV2e	KT795287	United States	PCV2e

### PCV2 Identity, Cells, and Viruses

*Spodoptera frugiperda* (Sf9) cells (ExpiSf9; Gibco) were cultured in Sf-900™ II SFM medium (Gibco) at 28 °C. PCV2b (GenBank accession number: FJ598044) was used for amplification of the nucleotide sequence of the recombinant Cap protein by PCR. The PCV2d-based Cap protein nucleotide sequence came from the PCV2d (HB16 strain). PK-15 cells without PCV infection were cultured in a modified serum-free medium (Celkey®CDPK15, China) and cultured at 37 °C under 5% CO_2_, which was used to propagate PCV2b and PCV2d.

### Construction of RBac-2bCap and RBac-2dCap

The recombinant baculovirus of PCV2b and PCV2d Cap proteins were constructed by the Bac-to-Bac Baculovirus Expression System (Invitrogen, USA) following the manufacturer's instructions. In short, the Cap protein genes of PCV2b and PCV2d were amplified with PrimeSTAR HS DNA polymerase (Takara, Japan). Primers are shown in Table 1. The first PCV2 Cap gene amplified by the F3/R3 primers was digested with BamHI and SacI (Takara, Japan) and cloned into the pFastBacDual vector (Invitrogen) containing the baculovirus polyhedrin gene promoter. The second PCV2 Cap gene amplified by the F4/R4 primers was digested with KpnI and NcoI and cloned into the downstream position controlled by the P10 promoter. Recombinant pFBD-2b Cap and pFBD-2d Cap were transformed into chemically competent *Escherichia coli* DH10Bac cells (Invitrogen). Recombinant bacmids were screened by blue and white spots and further verified by sequencing (Sangon, Wuhan, China). The recombinant bacmid DNA was transfected into Sf9 cells using CellfectinII reagent (Invitrogen), and rBac-2bCap and rBac-2dCap were generated. The recombinant baculovirus titers were computed by immunofluorescence assay.

### Expression and Purification of PCV2b and PCV2d Cap Proteins

Sf9 cells were infected with rBac-2bCap and rBac-2dCap in the MOI of 0.5 and then harvested at 7 days post-infection (dpi). The expressed recombinant Cap proteins were purified by anion exchange chromatography columns. In short, at 7 dpi, the harvested culture mixture was centrifuged at 10,000 × *g* for 30 min under 4 °C. Then, the supernatants were passed through a 0.45 μm polyethersulfone syringe filter. The harvested supernatants were purified by the Q Sepharose Fast Flow Ion Exchange Chromatography Columns (GE Healthcare, USA). The purified sample was assembled into virus-like particles (VLPs), which were observed under a transmission electron microscope (TEM; Hitachi, Japan) at the Huazhong Agricultural University.

### Sodium Dodecyl Sulfate-Polyacrylamide Gel Electrophoresis (SDS-PAGE) and Western Blot Analysis

The purified PCV2b and PCV2d Cap proteins were subjected to 12% SDS-PAGE and then transferred to nitrocellulose membranes (Merck, Germany) using semidry transfer apparatus (Bio-Rad). A porcine anti-PCV2 antibody (diluted 1:500) was used to prove the purified protein by western blot, and horseradish peroxidase (HRP)-conjugated goat anti-porcine IgG antibody (diluted 1:5,000) was the secondary antibody.

### Animal Experimental Design

Seventy 8-week-old female BALB/c mice were randomly divided into the following seven groups with 10 mice per group: phosphate-buffered saline (PBS), UnVac-2b/Ch, Vac2b-2b/Ch, Vac2d-2b/Ch, UnVac-2d/Ch, Vac2b-2d/Ch, and Vac2d-2d/Ch groups. At 28 days post-challenge (dpc, 8 weeks of age), 18 μg of purified PCV2b Cap protein was mixed with Montanide^TM^ ISA 201 VG (Seppic, France) adjuvant at a ratio of 1:1 (w/w) and then injected into the leg muscles of the BALB/c mice. Similarly, the immunization dose of the PCV2d Cap protein was the same as that of PCV2b. at −14 dpc, the second immunization was performed, the dosage and method of protein immunization were the same as those of the first immunization. At 0 dpc, 500 μL of PCV2 virus solution was injected intraperitoneally, and the BALB/c mice in the negative control group were injected with 500 μL of PBS. At 0 and 14 dpc, blood samples were collected and stored at −80 °C for the following experiments.

### Safety of Animal Immunization

Thirty 8-week-old female BALB/c mice were randomly divided into three groups (PBS, Vac PCV2b Cap, and Vac PCV2b Cap groups) and the 10**×** protein immunization dose (180 μg of purified protein) was injected into the mice to evaluate the safety of the vaccine. The clinical symptoms of the mice were monitored daily in the following 14 days after immunization. In short, the score was defined as follows: 0 (normal), 1 (rough haircoat), 2 (rough haircoat and dyspnea), 4 (severe dyspnea and abdominal breathing), and 6 (death). The observer was unaware of the different vaccination statuses of the mice.

### Enzyme-Linked Immunosorbent Assay (ELISA)

An indirect ELISA was used to test the PCV2-specific antibodies of 0 dpc in the serum samples. In short, a 96-well microtiter plate was coated with 50 ng/well of purified Bac-2b Cap or Bac-2d Cap protein at 4 °C overnight. Each well was washed with PBS 0.05% Tween 20 (PBS-T), then 200 μ L of PBS solution containing 2% bovine serum albumin was added to each well and incubated at 37 °C for 2 h. Subsequently, the plate was washed with PBS-T and 100 μ L aliquots of diluted serum samples (1:100) were added to each well and then incubated at 37 °C for 2 h. After washing with PBS-T, the plate was incubated with HRP-conjugated goat anti-mouse IgG (1:10,000; SouthernBiotech, USA) at 37 °C for 1 h. Subsequently, the plate was washed with PBS-T and TMB one-component HRP microwell substrate (SouthernBiotech, USA) was added, followed by incubation of the wells in the dark for 5 min. Finally, the reaction was stopped with 2N H_2_SO_4_, and then the OD450 nm value was determined by a microplate reader (Thermo Fisher Scientific, USA).

### Detection of PCV2 Viremia by PCR

The F1 and R1 primers were used to detect PCV2 and to amplify the PCV2 DNA in serum at 14 dpc. The experimental method was the same as the identification PCR experiment method. Primers are shown in Table 1.

### Detection of PCV2 Viremia by QPCR

The 14 dpc blood samples were collected and used to extract viral DNA by using a viral DNA/RNA extraction kit (TransGen, China) according to the manufacturer's instructions. Then, the concentration of viral nucleic acid was determined by a Nanodrop spectrophotometer. Then, qPCR was used for amplification using PCV2 ORF2-specific primers (PCV2_ORF2_F and R) and probing with PerfectStart™ II Probe qPCR SuperMix (TransGen, China) as shown in Table 1. The number of PCV2 DNA copies was calculated.

### Cytokine Assay

The 14 dpc sera were analyzed using mouse interferon-gamma (IFN-γ) ELISA kits (Multisciences, China) according to the manufacturer's instructions. The mean values of the cytokines were expressed as p g/m L.

### Statistical Analysis

Data were analyzed using one-way analysis of variance with Dunnett's multiple comparisons test using GraphPad Prism 7 software (GraphPad Software, USA). *p* values < 0.05 were considered to indicate significance.

## Results

### Prevalence of PCV2

**Figure 1:**
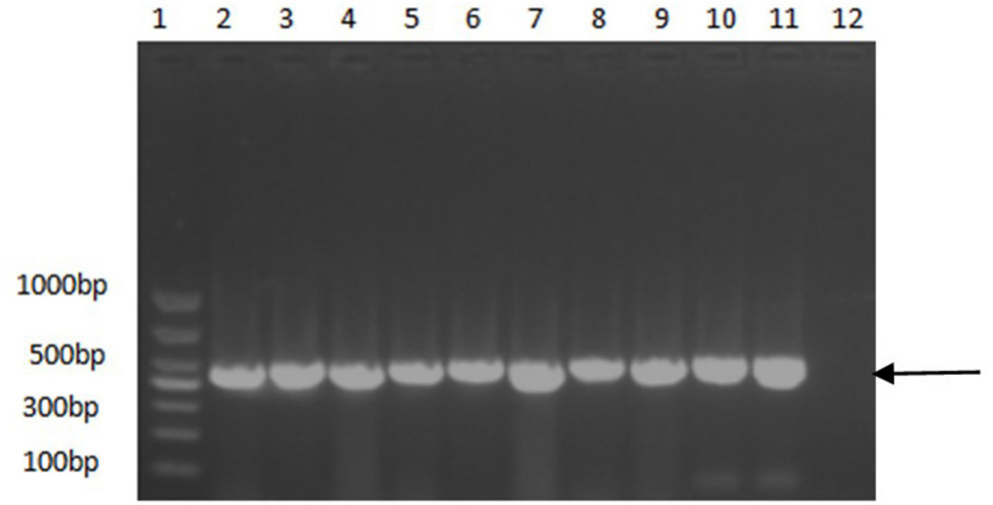
PCR identification results of some positive samples. 1, DNA molecular size marker; 2–10, Partial positive samples; 11, Positive control; 12, Negative control. Arrow indicates the position of the predicted DNA size.

A PCR assay was used to detect the PCV2 in the samples. As shown in Figure 1, the positive samples were able to amplify a band of about 496 bp. As shown in Table 3, among the 6,827 clinical pig samples collected from different pig farms in China and tested by PCR from 2018 to 2020, PCV2 was identified in 53% of them. At the sample level, the prevalence rate of PCV2 decreased continuously from 2018 to 2020; the positive rate in 2018 was 65.8% (2,379/4,164), in 2019 was 41.9% (450/1,071), and in 2020 was 26.2% (430/1,637). However, the unbalanced number of samples submitted each year may affect the positive rate.

**Table 3:** Temporal prevalence rates of PCV2 in China during 2018 to 2020.

**Region**	**Number of samples**	**Number of positive samples**	**Positive rate**
2018	4,164	2,739	65.8%
2019	1,071	450	41.9%
2020	1,637	430	26.2%
total	6,827	3,619	53%

### Sequence Analysis

**Figure 2:**
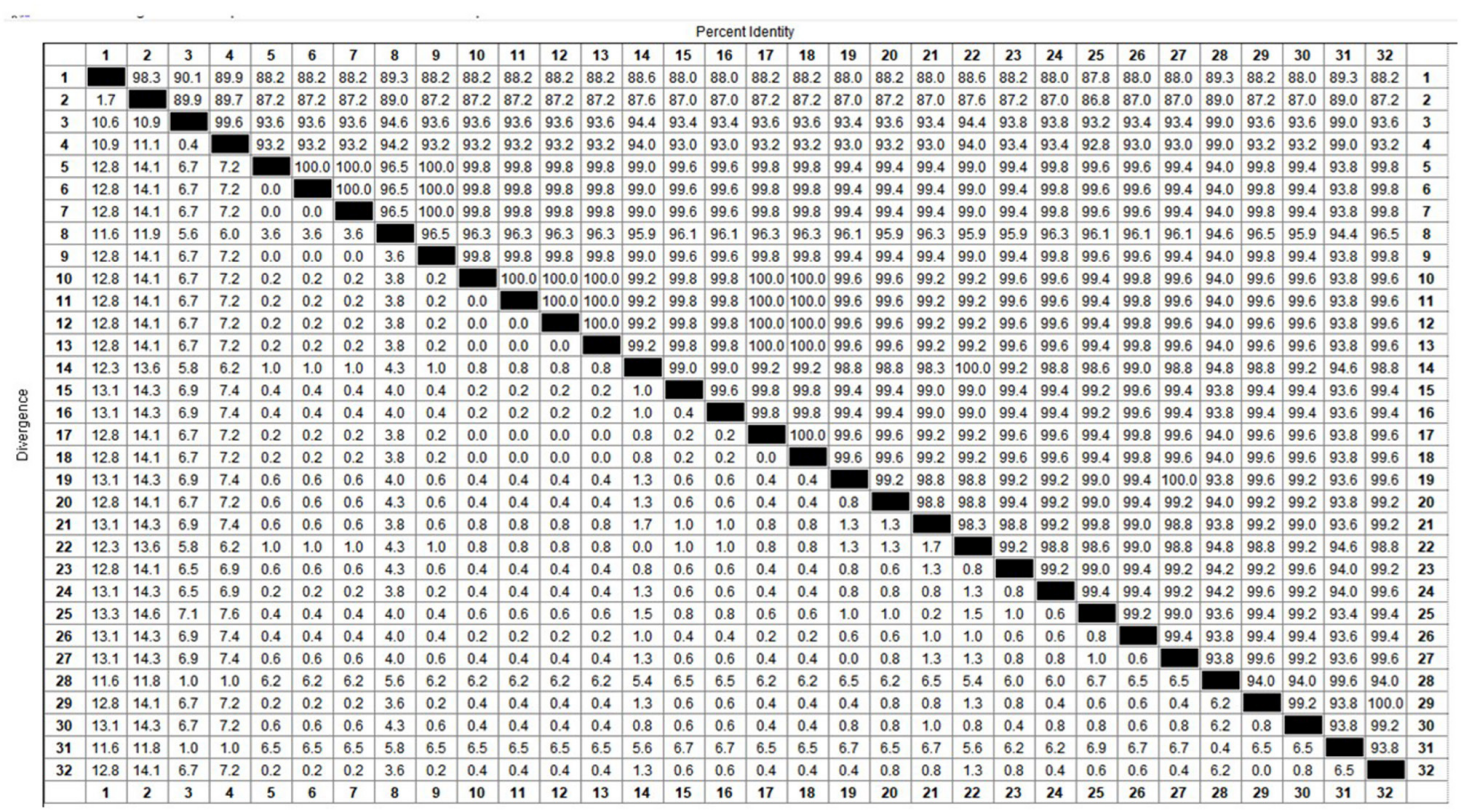
Identity analysis of partial sequences from PCV2 ORF2 gene.

**Figure 3:**
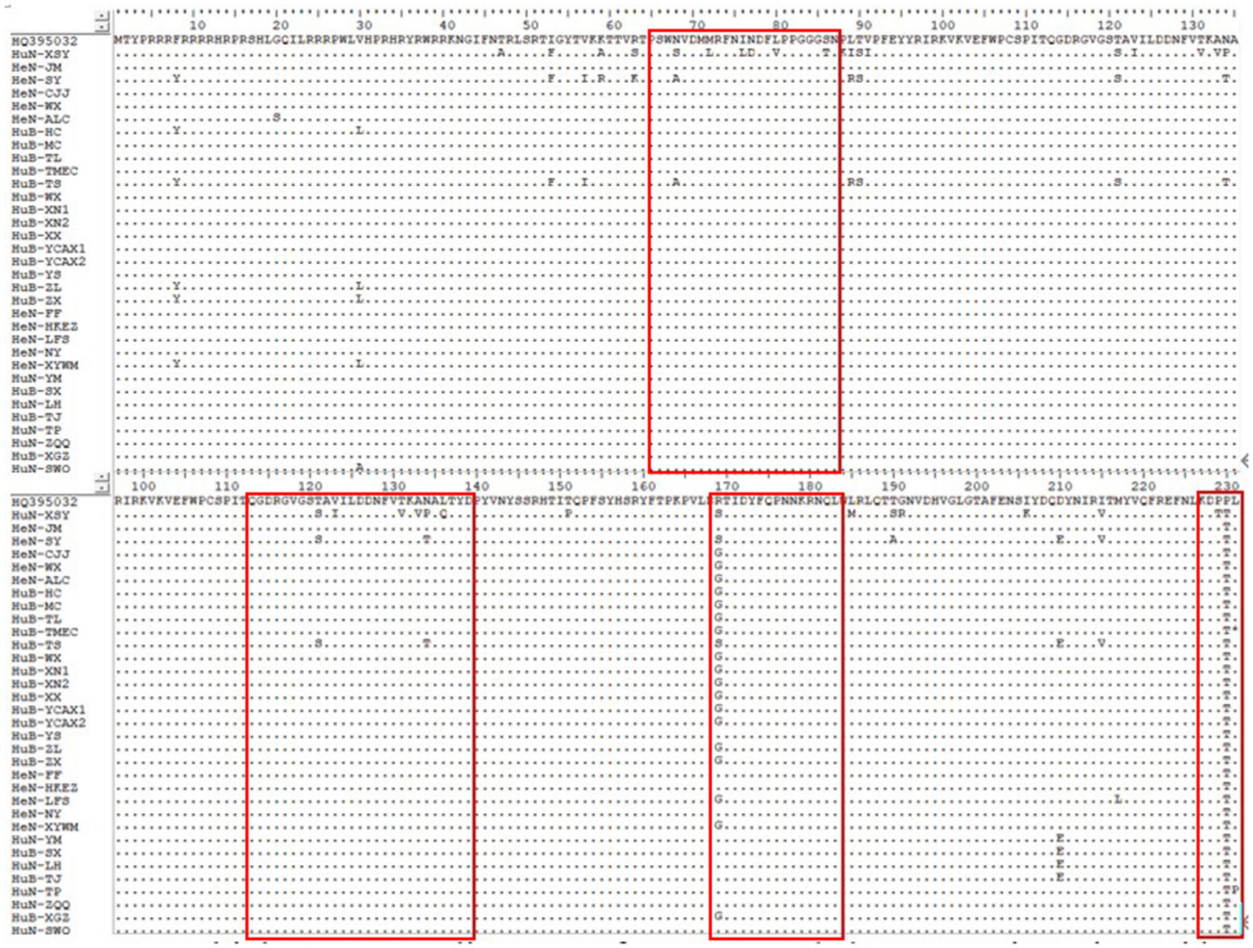
Multiple sequence alignment of ORF2-encoded Cap protein amino acids of some strains. Epitopes are boxed.

**Figure 4:**
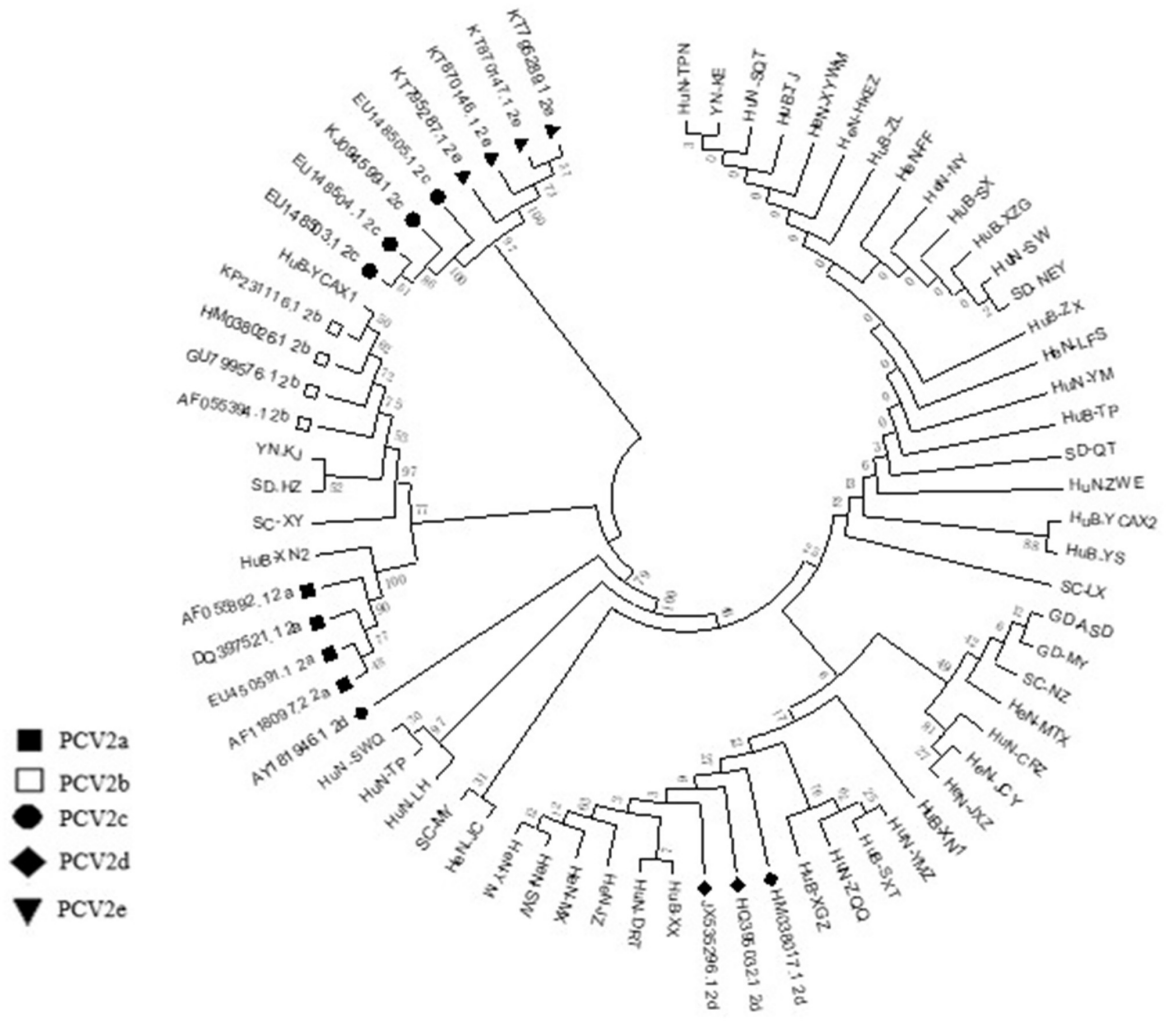
Phylogenetic tree based on 82 PCV2 nucleotide sequences, including the 62 samples obtained in this study (not marked) and 20 strains of the different genotypes available from GenBank (PCV2a to PCV2e) (marked). The phylogenetic tree was constructed by the neighbor-joining method using the maximum composite likelihood model with MEGA v6 software.

Among the collected samples, 62 with brighter PCR bands were selected for the cloning of the PCV2 ORF2 gene sequence and further sequence analysis. Through identity analysis of the nucleotide sequences by MegAlign software, we found that the identity of the ORF2 nucleotide sequences of 62 strains was 84.4–100%, and that of 62 strains with 20 reference sequences was 72.9–99.8%. The identity comparison results of some strains are shown in Figure 2. Multisequence alignment of the 62 amino acid sequences encoded by ORF2 genes was conducted using HQ395032 as the reference sequence. Although the 62 ORF2-encoded Cap protein sequences were relatively conservative (Figure 3), there were some scattered variable amino acid positions, such as 8, 30, 169, 210, and 230 aa. The Cap protein sequence encoded by HuN-XSY, HeN-SY, and HuB-TS strains were different at the epitope. This may affect the infectivity of the virus and the activity of neutralizing antibodies. Overall, 62 PCV2 isolate strain sequences were compared with 20 PCV2 reference strains, and the phylogenetic tree was constructed by MEGA V6.0 software. As shown in Figure 4, it was concluded that PCV2d accounted for 79% (49/62 samples), PCV2a for 12.9% (8/62 samples), PCV2b for 8% (5/62 samples), and PCV2c and PCV2e genotypes were not found, which proves that the PCV2d genotype was the epidemic strain of PCV2 in China from 2018 to 2020.

### Expression and Purification of Recombinant Protein

**Figure 5:**
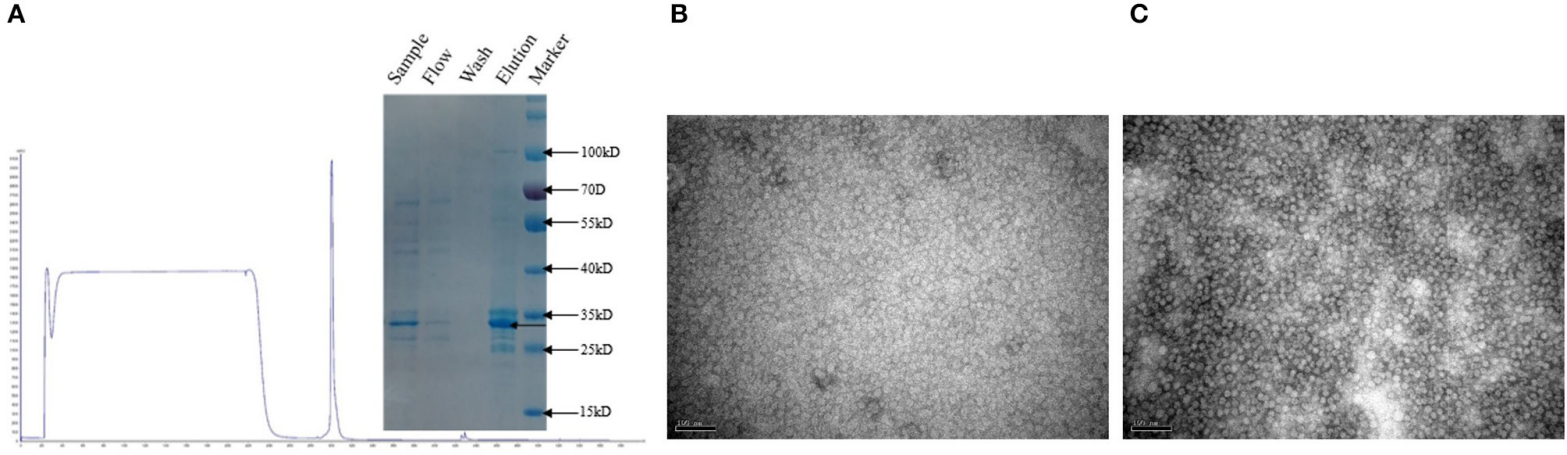
Purified sample subjected to SDS-PAGE **(A)**. TEM images of Bac-2bCap **(B)** and Bac-2dCap **(C)**.

The recombinant PCV-2b and PCV-2d Cap proteins were expressed in Sf9 cells and purified by anion exchange chromatography (Figure 5A). Purified Bac-2b and Bac-2d Cap proteins were successfully self-assembled into VLPs and were observed by TEM. As shown in Figures 5B,C, the assembled VLPs showed the expected particle size and shape with a diameter of 17 nm.

### Safety of the PCV2b and PCV2d Cap Protein Immunization in Mice

The safety of the PCV2b and PCV2d Cap proteins was evaluated in mice. Clinical monitoring records and scoring results are shown in Table 4. No notable clinical signs and no local and systemic adverse reactions were observed in any of the groups throughout the study. This shows that these two proteins are safe for mice. The PCV2b Cap protein induces protective immunity in mice against challenge with PCV2b and PCV2d strains.

**Table 4:** The clinical symptoms of the mice are scored.

**Group**	**Clinical score**					**Total**
	**0**	**1**	**2**	**4**	**6**	
PBS	10	0	0	0	0	10
Vac PCV2b cap	10	0	0	0	0	10
Vac PCV2d cap	10	0	0	0	0	10

*The score is defined as follows: 0 (normal), 1 (rough haircoat), 2 (rough haircoat and dyspnea), 4 (severe dyspnea and abdominal breathing), and 6 (death) 0 (normal), 1 (rough haircoat), 2 (rough haircoat and dyspnea), 4 (severe dyspnea and abdominal breathing), and 6 (death)*.

### PCV2b Cap Protein Induces Protective Immunity in Mice Against Challenge With PCV2b and PCV2d Strains

**Figure 6:**
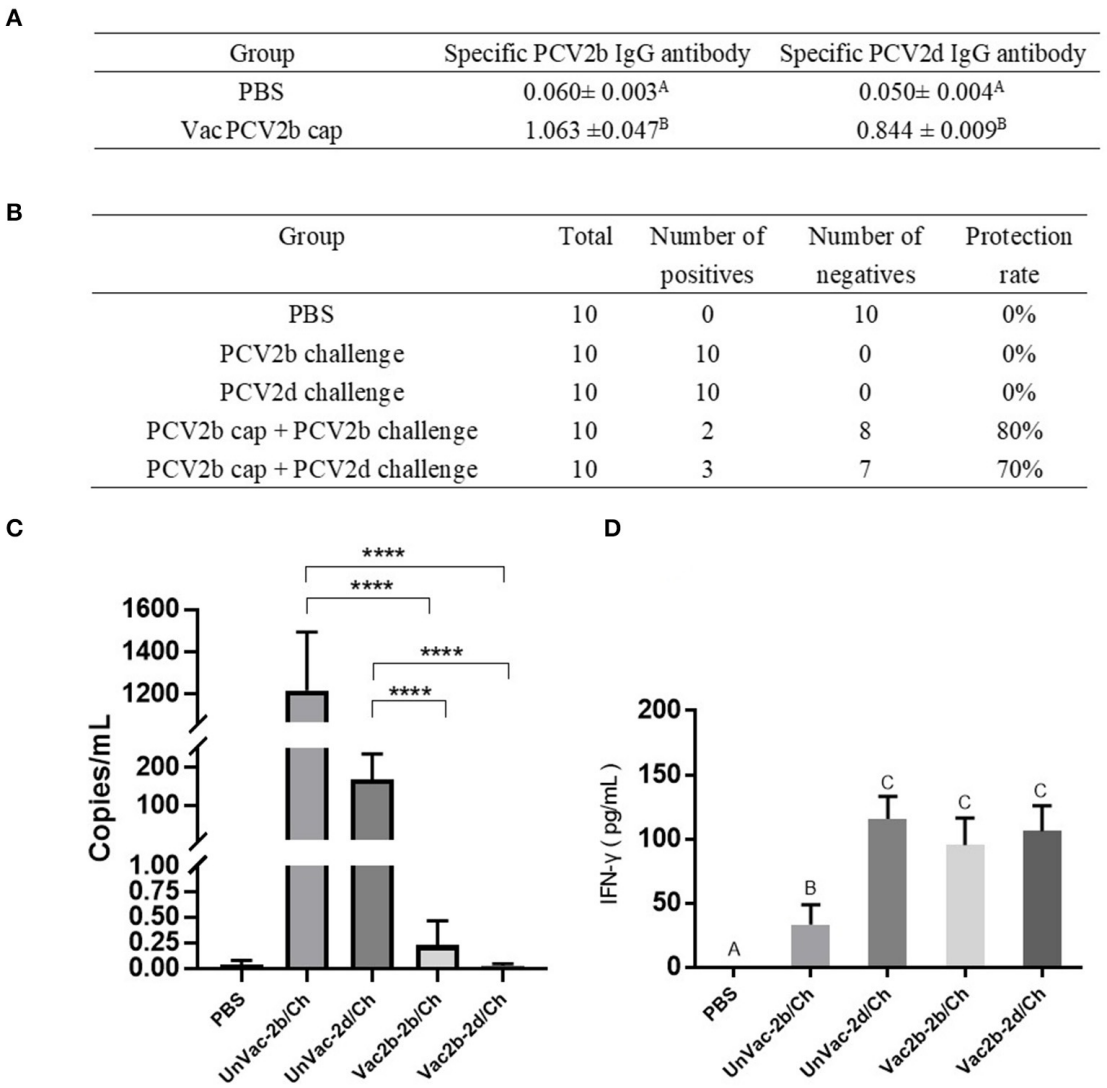
Homologous and heterologous immune response of PCV2d Cap protein against the PCV2b and PCV2dagainst the PCV2b and PCV2d. **(A)** Specific antibody levels for PCV2b and PCV2d Cap proteins at 0 dpc. **(B)** Detection of PCV2 DNA in serum by PCR at 14 dpc. **(C)** Detection of the PCV2 DNA load in serum by RT-PCR at 14 dpc. **(D)** Levels of IFN-γ in blood at 14 dpc. The data are presented as group mean optical density (M_OD_ ± standard error). Significant differences are indicated by different superscripts **(A–C)** (*p* < 0.05). Data were analyzed using one-way analysis of variance with Dunnett's multiple comparisons test using GraphPad Prism 7 software (GraphPad Software, USA), ^****^p < 0.001.

To evaluate whether the PCV2b Cap protein can protect pigs against infection with both PCV2b and PCV2d viruses, first, at 0 dpc, the PCV2b and PCV2d specific antibody levels in serum were determined by indirect ELISA using the Bac-2b and Bac-2d Cap antigens as a coating buffer. As shown in Figure 6A, VacPCV2b Cap maintained high levels of the anti-PCV2b- and the anti-PCV2d-specific IgG antibody. The anti-PCV2b-specific IgG antibody showed a mean OD value of 1.063, which was higher than that for the anti-PCV2d-specific IgG antibody at 0.844. The OD values of these were 17.69 (*p* < 0.001) and 16.88 (*p* < 0.001) times higher than that of the PBS group, respectively. Secondly, the 10 serum samples at 14 dpc were used to amplify PCV2 DNA by PCR (Figure 6B). The PCV2b and PCV2d challenge groups were all able to amplify positive bands, while Vac2b-2b/Ch showed a 2/10 and Vac2b-2d/Ch showed a 3/10 positive rate, showing that the PCV2b Cap protein can effectively resist the viremia of PCV2b and PCV2d. Thirdly, qPCR was performed using PCV2-specific primers for calculating the number of genomic DNA copies in the serum after the challenge (Figure 6C). Compared with the UnVac-2b/Ch group, the PCV2b viral load of the Vac2b-2b/Ch group decreased by 5,318 times, and the difference was significant (*p* < 0.001). Compared with the UnVac-2d/Ch group, the PCV2d viral load of the Vac2b-2d/Ch group decreased by 6,023 times, and the difference was significant (*p* < 0.001), which further verifies that PCV2b Cap protein is able to resist viremia caused by PCV2b and PCV2d. To evaluate whether the Bac-2bCap protein would elicit cellular immune responses, we analyzed the production of IFN-γ in serum (Figure 6D). At 14 dpc, high levels of IFN-γ were detected in all groups compared with the PBS group (*p* < 0.001). The UnVac-2b/Ch and UnVac-2d/Ch groups had slightly higher cytokine levels than the Vac2b-2b/Ch and Vac2b-2d/Ch groups, and the difference was not obvious, which indicated that PCV2b Cap protein stimulates TH1 cells to secrete IFN-γ cytokines. These results indicate that the PCV2b Cap protein can effectively activate the host's humoral and cellular immunity, thereby reducing the body's viremia caused by PCV2b and PCV2d.

### PCV2d Cap Protein Induces Protective Immunity in Mice Against Challenge With PCV2b and PCV2d Strains

**Figure 7:**
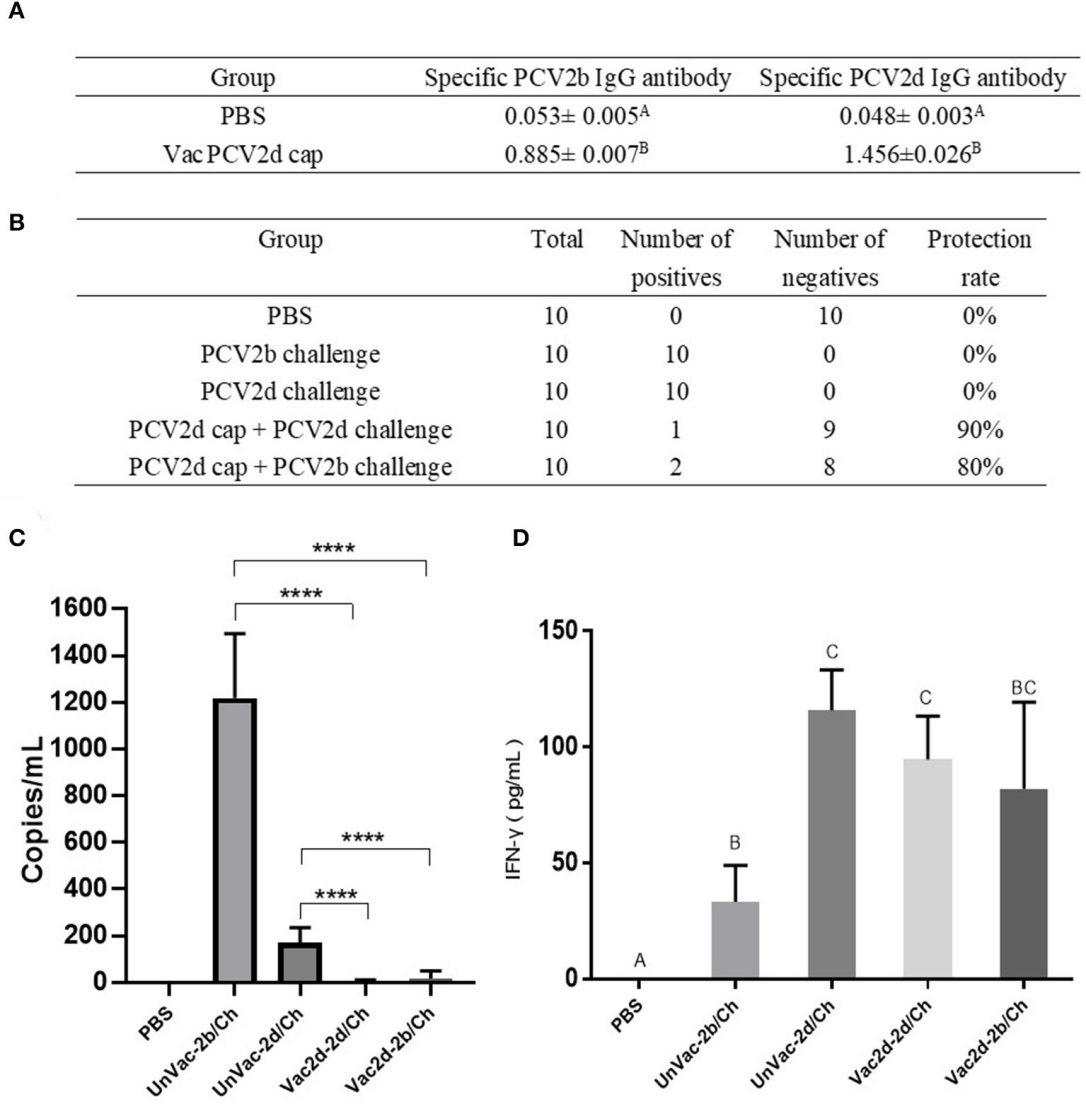
Homologous and heterologous immune response of PCV2d Cap protein against the PCV2b and PCV2dagainst the PCV2b and PCV2d. **(A)** Specific antibody levels for PCV2b and PCV2d Cap proteins at 0 dpc. **(B)** Detection of PCV2 DNA in serum by PCR at 14 dpc. **(C)** Detection of the PCV2 DNA load in serum by RT-PCR at 14 dpc. **(D)** Levels of IFN-γ in blood at 14 dpc. The data are presented as group mean optical density (M_OD_ ± standard error). Significant differences are indicated by different superscripts **(A–C)** (*p* < 0.05). Data were analyzed using one-way analysis of variance with Dunnett's multiple comparisons test using GraphPad Prism 7 software (GraphPad Software, USA), ^****^p < 0.001.

To evaluate whether the PCV2d Cap protein can protect pigs against infection with both PCV2b and PCV2d viruses, first, at 0 dpc, the PCV2b- and PCV2d-specific antibody levels in serum were determined by indirect ELISA using the Bac-2b and Bac-2d Cap antigens as a coating buffer. As shown in Figure 7A, the VacPCV2b Cap group preserved high levels of the anti-PCV2b-2d-specific IgG antibody and the anti-PCV2d-specific IgG antibody with a mean OD value of 1.456, which was 30.33 (*p* < 0.001) times higher than that of the control group, and the anti-PCV2b-specific IgG antibody with a mean OD value of 0.885, which was 16.69 (*p* < 0.001) times higher than that in the control group. Secondly, at 14 dpc, serum samples from mice were used to detect PCV2 DNA by PCR. The unvaccinated group with the challenge by PCV2b and PCV2d showed a 10/10 positive rate for both, while Vac2d-2b/Ch showed a 2/10 and Vac2d-2d/Ch a 1/10 positive rate, which proved that the PCV2d Cap protein can effectively reduce the viremia of both PCV2b and PCV2d (Figure 7B). Thirdly, we assessed the levels of the virus in the sera of infected mice by qPCR (Figure 7C). Compared with the UnVac-2b/Ch group, the PCV2b viral load of the Vac2d-2b/Ch group decreased by 71 times, and the difference was significant (*p* < 0.001). Compared with the UnVac-2d/Ch group, the PCV2d viral load of the Vac2d-2d/Ch group decreased by 42 times, and the difference was significant (*p* < 0.001). To further evaluate the T cell immune response stimulated by our PCV2d Cap protein vaccine candidate, we analyzed the production of IFN-γ in serum (Figure 7D). At 14 dpc, compared with the control group, mice from the experimental groups had high levels of IFN-γ (*p* < 0.001). UnVac-2b/Ch had significantly less IFN-γ cytokine levels than UnVac-2d/Ch (*p* < 0.05), which may be caused by the difference in the challenge dose. These results indicate that PCV2d Cap protein can effectively activate the host's humoral and cellular immunity, thereby resisting the body's viremia caused by PCV2b and PCV2d.

## Discussion

Since the discovery of porcine circovirus type 2 (PCV2) in 1998 (19, 20), PCV2 has gradually spread worldwide and caused huge economic losses to the global swine industry. In recent years, epidemiological surveillance results have shown that the PCV2 infection rate is on the rise in China (21). In addition, many studies have reported that the co-infection of PCV with other porcine pathogens, such as porcine reproductive and respiratory syndrome virus, porcine parvovirus, swine influenza virus, *Mycoplasma hyopneumoniae*, and salmonella may aggravate PCV2 infection, resulting in serious cases of PCVD.

This study showed that the positive rate of PCV2 was 53% (3,619/6,827 samples) in China from 2018 to 2020, which is close to the 50.3% of Northeast China from 2015 to 2018 (46). Moreover, we found that the identity of the ORF2 gene between 62 PCV2 isolates in this study was 84.4–100%, indicating that there are differences among PCV2 molecules prevailing in China. Cap protein encoded by the ORF2 gene is the main structural protein and key epitope cluster of PCV2. Compared with the reference PCV2d Cap protein amino acid sequence (GenBank accession number: HQ395032.1), the 62 isolates had higher variations in several regions, and there were some scattered variable amino acid positions, such as 8, 30, 169, 210, and 230 aa. Changes in the amino acid sequence of different strains may reveal differences in virulence, which will bring some difficulties to the prevention and control of PCV2 disease. The immunodominant epitope on the amino acid Cap protein of PCV2 is recognized by the antibodies of PCV2-infected pigs. It is characterized by different A, B, C, and D regions, which roughly correspond to the amino acid fragments 65–87, 113–139, 169–183, and a few C-terminal amino acids (22, 23), respectively. the Cap protein sequence encoded by HuN-XSY, HeN-SY, and HuB-TS strains were variable at the epitopes A, B, and C, which may affect the effectiveness of antibodies produced by the vaccine.

Studies have reported that the PCV2d genotype has become the predominant genotype in some regions of China, including Yunnan province, Hunan province, Shandong province, and northwest China (24–28). The positive rate of the PCV2d genotype was as high as 79.0% in this study, indicating that PCV2d has been the prevalent strain in China. With the discovery of PCV2d and subsequent genotype shift from the previously predominant PCV2b to PCV2d, concerns over PCV2 vaccine efficacy have been raised. Although all current vaccines have been proven to be effective at preventing clinical signs and global economic loss due to PCVAD (29, 30), the emergence of the PCV2d subtype cannot be ignored (31). A study pointed out that the PCV2b-based Cap protein expressed by baculoviruses can induce the body to produce greater humoral and cellular immunity, and this subunit vaccine can effectively reduce the viremia of pigs naturally infected with PCV2d (32). Logically, PCV2d Cap protein can provide better protection against PCV2d than PCV2b Cap protein. Therefore, the main purpose of this study was to explore the protection ability of the PCV2b and PCV2d Cap proteins against the PCV2b and PCV2d virus challenge.

In this study, after immunizing twice, the PCV2b and PCV2d Cap protein immune groups both contained higher levels of PCV2b and PCV2d IgG antibodies, and the titer of antibodies against the PCV2d Cap protein was slightly higher than that against the PCV2b Cap protein, but this difference was not significant.

PCV2 infection can cause severe viremia (31). PCR detection of PCV2 DNA in serum samples at 14 dpi found that the positive rate of UnVac-2b/Ch and UnVac-2d/Ch viral DNA reached 100%. However, the positive rates of viral DNA in the Vac2b-2b/Ch and Vac2b-2d/Ch groups were 20 and 30%, respectively, and those of viral DNA in the Vac2d-2b /Ch and Vac2d-2d/Ch groups were 20 and 10%, respectively.

Compared with the 2b/Ch and 2d/Ch groups, the viral load of PCV2b and PCV2d in the Vac2b group decreased by 5,318 and 6,023 times, respectively, while the viral load of PCV2b and PCV2d in the Vac2d group decreased by 71 and 42 times, respectively. This shows that both proteins can reduce the virus infection of the body and, under the same protein immune dose, the effect of the PCV2b Cap protein in reducing viremia is better than that of the PCV2d protein. This is inconsistent with the results of conventional PCR. Conventional PCR can only be qualitative but not quantitative, and the criteria for judging negative and positive have certain subjective characteristics, while RT-PCR can perform absolute quantification of viral DNA, and its results will be more reliable than the former.

Increased secretion of IFN-γ is responsible for the reduction of PCV2 viremia (33, 34), where the level is considered the measurement of protective immunity (35, 36). The UnVac-PCV2b/Ch group had more PCV2 genome copy numbers than the UnVac-PCV2d/Ch group, while the levels of IFN-γ of the UnVac-PCV2b/Ch group were lower than the latter, which is consistent with the previous conclusion. Compared with the blank control group, the levels of IFN-γ were higher in all experimental groups, indicating the improvement of the IFN-γ secretion level induced by the TH1 cells after immunization with protein.

In summary, PCV2d was found to be the current circulating strain in pigs in China. PCV2b and PCV2d Cap proteins expressed by the baculovirus system can both stimulate the level of humoral immunity and cellular immune response in mice. Interestingly, these two proteins had certain homologous and heterologous immune response against the PCV2b and PCV2d strains, and the effect of the PCV2b Cap protein in reducing viremia was better than that of the PCV2d Cap protein. Therefore, this study has significance for the subsequent prevention and control of PCV and the preparation of a suitable vaccine.

## Data Availability Statement

The original contributions presented in the study are included in the article/supplementary materials, further inquiries can be directed to the corresponding author/s.

## Ethics Statement

The animal study was reviewed and approved by Animal Experimental Ethical Inspection of Laboratory Animal Centre, Huazhong Agriculture University.

## Author Contributions

YH designed and performed the experiments, analyzed the data, and wrote the manuscript. XC, YL, LY, WS, JL, QL, GL, and DY performed the experiments. CH and XT conceived the project, analyzed the data, and revised the manuscript. All authors contributed to the article and approved the submitted version.

## Funding

This study received funding from Wuhan Keqian Biology Co., Ltd. The funder was not involved in the study design, collection, analysis, interpretation of data, the writing of this article, or the decision to submit it for publication.

## Conflict of Interest

All authors are employed by Wuhan Keqian Biology Co., Ltd.

## Publisher's Note

All claims expressed in this article are solely those of the authors and do not necessarily represent those of their affiliated organizations, or those of the publisher, the editors and the reviewers. Any product that may be evaluated in this article, or claim that may be made by its manufacturer, is not guaranteed or endorsed by the publisher.
